# Diagnostic performance of diffusion-weighted imaging for prostate cancer: Peripheral zone versus transition zone

**DOI:** 10.1371/journal.pone.0199636

**Published:** 2018-06-22

**Authors:** Hakmin Lee, Sung Il Hwang, Hak Jong Lee, Seok-Soo Byun, Sang Eun Lee, Sung Kyu Hong

**Affiliations:** 1 Department of Urology, Seoul National University Bundang Hospital, Seongnam, Korea; 2 Department of Radiology, Seoul National University Bundang Hospital, Seongnam, Korea; 3 Department of Radiology, Seoul National University College of Medicine, Seoul, Korea; 4 Department of Urology, Seoul National University College of Medicine, Seoul, Korea; University of Chicago, UNITED STATES

## Abstract

**Objectives:**

Diffusion-weighted imaging (DWI) has been shown to be an important component of multiparametric magnetic resonance imaging (mpMRI). We compared performance of DWI for detection of prostate cancer (PCa) in peripheral zone (PZ) and transition zone (TZ) of prostate.

**Materials and methods:**

We reviewed data of 460 subjects who underwent preoperative 3.0-Tesla mpMRI and subsequently radical prostatectomy. Level of suspicion for PCa was graded using 5-grade Likert-scale from DWI. Topographic analyses were performed for location of tumor foci at each surgical specimen. Among those with DWI grade ≥ III, we analyzed concordance rate on the location of radiologic and pathologic index lesions between DWI and surgical specimens.

**Results:**

Among 460 patients, 351 (76.3%) patients showed suspicious DWI lesions (57.5% in PZ, 42.5% in TZ). Multivariates regression analyses revealed significant associations between high DWI grade and adverse pathologic outcomes including pathologic stage, Gleason score, tumor volume and extracapsular extension (all p < 0.05). Overall concordance rates between DWI and surgical specimen were 75.8%, significantly higher in PZ than TZ (82.2% vs. 67.1% p = 0.002). Such concordance rate showed a positive linear association with increase in DWI grading (p < 0.001). Among 109 patients with DWI grade I-II, 28 (25.7%) harbored high grade disease (pathologic Gleason score ≥ 4 + 3).

**Conclusions:**

DWI detects tumors in PZ of prostate more accurately than those in TZ. Such accuracy of DWI was shown to be more evident with higher DWI grade. Meanwhile, a negative DWI did not guarantee absence of high grade PCa.

## Introduction

Today, multiparametric magnetic resonance imaging (mpMRI) has emerged as an accurate diagnostic tool for detection and grading of prostate cancer (PCa). Currently, American College of Radiology and European Society of Urogenital Radiology (ESUR) supports the use of mpMRI as the reference standard imaging for evaluation of the prostate [1–2]. Several studies have demonstrated that mpMRI can accurately identify dominant tumor foci, showing very high negative predictive value for the detection of clinically significant PCa [3–4].

Diffusion-weighted imaging (DWI), a cornerstone of functional prostate mpMRI, has been shown to be an important component of mpMRI, especially in the detection and grading of PCa [5]. DWI offers relatively shorter acquisition time, high contrast resolution between benign and malignant tissue, and apparent diffusion coefficient (ADC) maps which provide tumor location and estimate of tumor grade [6–7]. On the other hand, DWI has also been known to suffer from potentials for artifact [8]. Moreover optimal method for the measurement of ADC has yet to be determined. Although DWI is widely regarded as a relatively accurate imaging sequence for the evaluation of peripheral zone (PZ), opinions are mixed on the usefulness of DWI in the detection of PCa in transition zone (TZ) [7]. Diagnosis of TZ tumors by T2-weighted (T2W) imaging only is limited by heterogeneity of TZ in older men. Combining DWI with T2W has been shown to improve diagnostic performance. Meanwhile, although stromal benign prostatic hyperplasia (BPH) in TZ may have higher ADC than cancer tissue, use of quantitative ADC in the discrimination between BPH and TZ cancer needs further validation [9]. The Prostate Imaging-Reporting and Data system guidelines (Version 2) which was established for the standardizing the MRI acquisition and interpretation are suggesting the DWI as the primary assessment component in PZ but not in the TZ [10]. Furthermore, controversy continues on the value of dynamic contrast-enhanced (DCE) imaging of mpMRI in the detection of TZ cancer due to the microvessel density by BPH [11]. Currently, variable rates of accuracy of mpMRI have been reported in the detection of TZ or anterior PCa [7, 12]. Thus in this study, we tried to evaluate and compare the accuracies of DWI, a major component of mpMRI, in the detection of PCa in PZ and TZ of the prostate.

## Materials and methods

**Fig 1:**
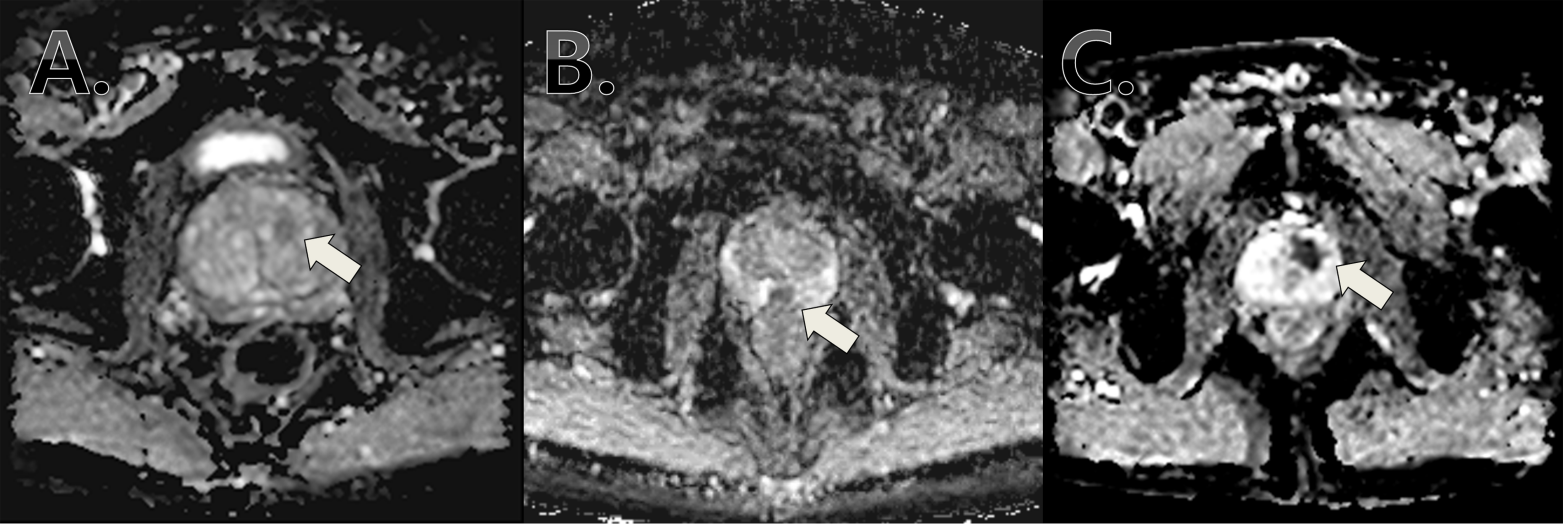
The examples of diffusion weighted images according to the Likert score; (A) grade III; (B) grade IV; (C) grade V.

After the approval from our institutional ethical review board (Seoul National University Bundang Hospital Institutional Review Board: B-1706/402-115), we analyzed the records of 460 consecutive patients who treated by radical prostatectomy (RP) for localized prostate cancer with preoperative mpMRI between January 2015 and May 2016. Clinico-pathologic information was retrieved from our prospectively maintained institutional database. mpMRI was performed using a 3.0 Tesla system (Philips Healthcare, Intera Achieva) using a phase-array coil without using endo-rectal coil [13]. Axial DWI was acquired using single-shot echo planar imaging technique and automatically generated on a pixel-by-pixel basis by the imaging software (repetition time, 3000ms; field of view, 220 x 220 mm; section thickness, 5mm; intersection gap, 1mm; matrix, 92 x 90; number of excitation, 10). Diffusion encoding gradients were applied as a bipolar pair at b-values 0 and 1,000 s/mm^2^ Two uro-radiologists who were blinded to clinical characteristics including pathologic outcomes, performed the interpretation of MRI using INFINITT PACS system (Infinitt healthcare, Seoul, South Korea) and RadiForce GS550 monitor (EIZO corporation, Japan) in the ambient light below 10 lux. They indepentently determined the level of suspicion of DWI map using the 5-grade Likert scale from I to V as the followings: grade I, highly unlikely; grade II, unlikely; grade III, equivocal; grade IV, likely; and grade V, highly likely to be present, as previously reported [13] (Fig 1). The decision of MRI suspicion grade was performed synthetically according to various aspect such as size of lesion (measured in the ADC map), brightness of signal and conformity with T2 images. Index lesion on MRI was defined as highest grade and/or the largest lesion when multiple lesions had an identical grade.

From analyzing RP specimens, topographic analyses were performed on the intraprostatic location of tumor foci. Intraprostatic locations of tumor foci were assessed as previously reported [14]. The pathologic index tumor was defined as the tumor with the highest Gleason score (GS) and/or largest tumor when multiple foci had an identical GS. As the patients with DWI grade ≤ II could not have index lesions, only patients with DWI grade ≥ III were evaluated in analyzing concordance between the pathologic and radiologic index lesions. When the location of pathologic index tumor assessed via previously-reported scheme was considered to correspond with the location of radiologic index tumor, the subjects were regarded to have the concordance of index lesions between preoperative MRI and surgical specimen [14]. High grade disease was defined as PCa having pathologic GS ≥ 4 + 3 from surgical specimens. Tumor volume was calculated as previously described [14–15]. Following RP, patients were usually followed up as 3 to 6 month intervals during the initial 2 years and yearly thereafter.

Chi-square and student T tests were performed to compare the characteristics between the groups. Multivariate regression tests were performed to evaluate possible associations of DWI findings with pathological outcomes. Following variables were included in the multivariate regression tests: age, body mass index, prostate volume, level of prostate specific antigen, biopsy Gleason score, and clinical stage. All the statistical analyses were performed using SPSS software (SPSS 19.0, Chicago, IL, USA). All p-values presented as two-sided and p < 0.05 was considered to be statistically significant.

## Results

Clinico-pathological characteristics of entire patients are summarized in Table 1. There were 109 (23.7%) patients who had no suspicious lesion (DWI grade ≤ II) in preoperative MRI and 351 (76.3%) patients with suspicious lesion (DWI grade ≥ III). Among the 351 patients (DWI grade ≥ III), 202 patients (58%) had index lesions on DWI observed in the PZ, and 149 patients (42%) in the TZ. The patients with suspicious lesion showed significantly unfavorable clinical characteristics including higher age, prostatic specific antigen (PSA), biopsy GS and clinical stage (all p values < 0.05). Furthermore, we also observed significantly worse pathologic outcomes in patients with suspicious lesion in DWI such as higher pathologic GS, pathologic stage, tumor volume, higher rate of extracapsular extension and seminal vesicle invasions (all p values < 0.05). The subsequent multivariates analyses also showed significant associations between higher DWI grading and unfavorable pathologic outcomes including having higher pathologic GS (≥ 4 + 3), higher pathologic stage (≥ 3), tumor volume (≥ 2.9cc) and extracapsular extension (all p values < 0.05) (Table 2).

**Table 1:** Clinicopathologic characteristics of subjects according to the suspicion level for prostate cancer determined from diffusion weighted imaging.

	No suspicious lesion (N. = 109)	With suspicious lesion (N. = 351)	p value
Age (y)	64.0 (59.0–70.0)	67 (61–72)	0.010
BMI (*kg*/*m*^2^)	24.2 (22.6–25.9)	24.5 (22.9–26.5)	0.583
PSA (ng/dl)	6.0 (4.1–8.5)	8.6 (5.8–13.0)	< 0.001
Prostate volume (g)	31.9 (25.5–38.2)	32.9 (26.4–40.5)	0.142
Biopsy GS			< 0.001
= 6	48 (44.0%)	79 (22.5%)	
= 7	58 (53.2%)	191 (54.4%)	
≥ 8	3 (2.8%)	81 (23.1%)	
Clinical stages			< 0.001
cT1	97 (21.1%)	217 (61.0%)	
cT2	11 (10.1%)	94 (26.8%)	
≥cT3	1 (0.9%)	43 (12.3%)	
DWI grade			< 0.001
Grade I-II	109 (100%)		
Grade III-IV		189 (53.8%)	
Grade V		162 (46.2%)	
Pathologic GS			< 0.001
≤ 3 + 4	81 (74.3%)	141 (40.2%)	
= 4 + 3	26 (23.9%)	150 (42.7%)	
≥ 4 + 4	2 (1.8%)	60 (17.1%)	
Pathologic stages			< 0.001
pT2	100 (91.7%)	227 (64.7%)	
≥pT3	9 (8.3%)	124 (35.3%)	
Tumor volume (g)	1.6 (0.7–2.9)	3.5 (2.0–6.3)	< 0.001
ECE	8 (7.3%)	119 (33.9%)	< 0.001
SVI	3 (2.8%)	31 (8.8%)	0.035
PSM	12 (11.0%)	63 (17.9%)	0.102

N. = number, BMI = body mass index, PSA = Prostate specific antigen, GS = Gleason score, ECE = extracapsular extension, SVI = Seminal vesicle invasion, PSM = positive surgical margin

**Table 2:** Multivariate regression analyses on the potential associations of preoperative diffusion-weighted imaging grading on various pathologic outcomes.

End-points	Entire patients			
	Suspicion grade	HR	95% CI	p value
pGS ≥ 4 + 3	I-II	Reference		
	III-IV	1.523	0.844–2.748	0.162
	V	5.470	2.813–10.637	< 0.001
Pathologic stage (≥ 3)	I-II	Reference		
	III-IV	2.319	1.052–5.113	0.037
	V	5.908	2.651–13.165	< 0.001
Tumor volume (≥ 2.9 cc^3^)	I-II	Reference		
	III-IV	2.628	1.537–4.494	< 0.001
	V	5.608	3.053–10.301	< 0.001
ECE	I-II	Reference		
	III-IV	2.328	1.015–5.341	0.046
	V	6.082	2.636–14.035	< 0.001
SVI	I-II	Reference		
	III-IV	1.849	0.482–7.099	0.371
	V	2.254	0.563–9.028	0.251

HR = hazard ratio, CI = confidence interval, pGS = pathologic Gleason score, ECE = extracapsular extension, SVI = seminal vesicle invasion

All statistical analyses were controlled by following variables: age, body mass index, prostate volume, prostate specific antigen, biopsy Gleason score and clinical stage.

The overall concordance rate between the radiologic and pathologic index lesions showed positive linear association with the increase in DWI grading (grade III: 52.3%, grade IV: 71.7%, grade V: 85.8%; p < 0.001). When we compared the concordance rates between the PZ and TZ, PZ showed a significant higher concordance rate than TZ (82.2% versus 67.1%, p = 0.002) (Table 3). However the subsequent subgroup analyses showed that those difference was statistically significant only in grade V lesions (PZ: 91.3% versus TZ: 75.9%, p = 0.010). No significant difference was observed among the subgroup of patients with grade III lesions and grade IV lesions (all p values > 0.05). The incidences of high grade disease in prostatectomy specimen also showed significant linear positive relationship according to the increase of DWI grade (grade III: 29.5%, grade IV: 48.3%, grade V: 78.4%; p < 0.001). The incidence of high grade disease was significantly more prevalent in PZ group than TZ group (66.8% versus 50.3%, p = 0.002) (Table 3). As the diameter of index lesion of 2.0 cm showed maximal Youden’s score after analyzing the receiver operating curves upon high pathologic stage (≥3) and postoperative biochemical recurrence, we stratified the patients (DWI grade ≥ 3) according to diameter of index lesion (< 2 cm versus ≥ 2cm) on mpMRI. The concordance rates were significantly higher in PZ than TZ in men with relatively smaller (< 2cm) index lesion (85.7% versus 57.4%, p < 0.001). However, no such findings were observed among those with larger (≥ 2 cm) index lesion (p = 0.248). Meanwhile, among the 109 patients without suspicious lesions in DWI imaging (grade ≤ II), 28 (25.7%) patients were revealed to harbor high grade disease after RP.

**Table 3:** The concordance rates of index lesions between diffusion-weighted imaging and prostatectomy specimen and the incidences of high grade disease according to diffusion-weighted imaging grade.

	Concordance rates of index lesions between DWI and prostatectomy specimens			Incidences of high grade disease in prostatectomy specimen (pGS ≥ 4 + 3)		
	PZ (n = 202)	TZ (n = 149)	p value	PZ (n = 202)	TZ (n = 149)	p value
Overall (III ~ V)	166 (82.2%)	100 (67.1%)	0.002	135 (66.8%)	75 (50.3%)	0.002
Suspicion grade III	10 (50.0%)	13 (54.2%)	1.000	7 (35.0%)	6 (25.0%)	0.522
Suspicion grade IV	61 (78.2%)	43 (64.2%)	0.067	40 (51.3%)	30 (44.7%)	0.506
Suspicion grade V	95 (91.3%)	44 (75.9%)	0.010	88 (84.6%)	39 (67.2%)	0.016

pGS = pathologic Gleason score, PZ = peripheral zone, TZ = transition zone

## Discussion

In our study, we observed that DWI was more useful for the detection of PCa in PZ than TZ. Moreover, such difference in diagnostic accuracy was found to be more evident in patients with higher DWI grade. In addition, our findings also confirmed that negative DWI findings do not guarantee the absence of high grade PCa.

As about 70% to 75% of PCa arise from PZ of the prostate, appropriate evaluation of PZ lesions would be important. Findings from our study demonstrated that DWI performs better for lesions in PZ than those in TZ. DWI exploits random motion of water molecules which is impeded by interactions with tissue compartments and cell membranes [16]. It is widely known that in PCa tissue, ADC value is proportional to luminal space and inversely related to the percentage area of nuclei [17]. In PCa tissues of higher Gleason score, cellularity would be higher whereas cellularity would be less in PCa tissues of lower Gleason score. Thus, more cellular cancer tissue of higher Gleason score would show more restricted diffusion compared with less cellular cancer tissue of lower Gleason score. Meanwhile, a benign structure of high cellularity having restricted diffusion, such as BPH nodule, can contribute to a false-positivity in the diagnosis of PCa. As BPH predominantly occurs in TZ, potentials for such false-positivity of DWI would be higher in TZ. Such phenomenon may partly explain superior accuracy of DWI in PZ compared with TZ.

Most of BPH nodules demonstrate a mixed pattern with both glandular and stromal components [18]. Evaluation of TZ for PCa can be challenging in the presence of BPH as stromal BPH nodules can simulate tumors on mpMRI. Due to high percentage of muscular and fibrous components, stromal BPH nodules demonstrate low signal intensities on T2W images similar to PCa [19]. Stromal BPH nodules also demonstrate low signal intensities on ADC owing to inherently low signal intensities on T2W and true restricted diffusion due to dense cellularity. Despite the fact that stromal BPH demonstrated higher ADC than PCa, the use of quantitative ADC in the diagnosis of PCa in TZ still is yet to be validated [9].

In 2012, ESUR published the first version of the Prostate Imaging Reporting and Data System (PI-RADS) to standardize the report of mpMRI comprising T2W, DWI, and DCE sequences [2]. In PI-RADS version 1 system, individual score (based upon 5-point Likert scale) was assigned to each MRI sequence. Meanwhile, some have suggested that different weightings should be used depending on the location of the lesion (PZ versus TZ). Baur et al reported that assigning a PI-RADS score on the basis of DWI for PZ lesions and a PI-RADS score on the basis of T2W imaging for TZ lesions was sufficient for stratification of patients for further diagnostic workup [20]. In 2015, ESUR prostate MR expert group and PI-RADS steering committee of American College of Radiology came up with PI-RADS version 2 system supposedly to overcome the limitations of PI-RADS version 1. PI-RADS version 2 was developed to take the location and size of a lesion into consideration [10]. Schieda et al reported that PI-RADS version 2 decreases the differences in diagnostic accuracy regarding the radiologists’ experience than version 1 [21]. Kasel-Seibert et al compared PI-RADS version 1 and 2 in ≥ 3 lesions (based on version 1 criteria) and reported that version 2 had higher accuracy [22]. Recently, Polanec et al compared diagnostic performances of PI-RADS version 1 and 2 by analyzing 65 consecutive patients undergoing MR-guided biopsy after mpMRI [23]. They found that PI-RADS version 2 had higher sensitivity than version 1 in TZ. We believe that our findings can be supportive of PI-RADS version 2 as DWI was shown to be more accurate in the evaluation of PZ lesions in our study.

Despite our observation that DWI performs better in the evaluation of PZ lesions, DWI is by no means perfect. For instance, it is known that prostatitis lesions can present with equivocal DWI findings [23]. For these cases, T2W images are needed to minimize the risk of false-positivity. Even though our results can be considered supportive of the implementation of PI-RADS version 2, which utilizes DWI as primary sequence for PZ lesions, multi-parametric approach should still be practiced for the optimal performance of mpMRI. Others have shown that mpMRI has high negative predictive value for high-grade PCa with some reporting that mpMRI’s negative predictive value for high-grade PCa reached 100% among patients who were biopsy naïve or who were under active surveillance for Gleason 6 disease [24–26]. However, despite such reports of near-perfection from some researchers, others have reported to the contrary. From analyzing 122 patients who underwent mpMRI before RP, Le et al demonstrated that about 20% of index tumors were missed by mpMRI [27]. Also, in a series of 101 patients who underwent RP with a negative preoperative mpMRI, 61.4% were found to harbor tumors of pathologic GS ≥ 7 [28]. In our study, we also observed that a proportion of patients harbored high grade disease despite scoring Likert grade I-II on preoperative DWI. Looking at these findings, risk of having significant cancer despite a negative DWI should not be regarded negligible.

We acknowledge that there may be limitations to our study, including limited number of subjects and retrospective nature. As two radiologists analyzed the mpMRI, we admit some possibility for interobserver variability. However, two radiologists who participated in our study have more than 20-years of clinical experience each dedicated to the field of uro-radiology. For our study, we used grading system developed in-house instead of PI-RADS which limits the present study’s clinical applicability. Admittedly, usage of different grading system may have impacted the outcome. Also, possibility of selection bias towards having more significant cancer exists as we analyzed only those who underwent RP. However, it would be more appropriate to investigate the pathologic features from RP specimens rather than the biopsy findings to elucidate true pathologic outcomes of patients with negative DWI. Moreover, we could not analyze the clinical role of quantitative ADC in the present study, which is one of our main limitations.

## Conclusions

Our results demonstrated that DWI detects tumors in PZ more accurately than TZ. Among patients with highest Likert scale of 5, DWI proved to be more accurate in the detection of PCa in PZ than those in TZ whereas no significant difference was observed among patients with lower scale. Also non-negligible number of patients with a negative DWI was revealed to harbor high grade PCa. Such findings should be considered in the interpretation of mpMRI performed for the detection of PCa.
